# Research Progress on Natural Products' Therapeutic Effects on Atrial Fibrillation by Regulating Ion Channels

**DOI:** 10.1155/2022/4559809

**Published:** 2022-03-22

**Authors:** Jinshan He, Sicong Li, Yumeng Ding, Yujia Tong, Xuebin Li

**Affiliations:** ^1^Cardiovascular Department, Peking University People's Hospital, Beijing, China; ^2^School of Pharmacy, Peking University Health Science Centre, Beijing, China; ^3^School of Life Sciences, Shanxi Normal University, Shanxi, China; ^4^Institute of Medical Information, Chinese Academy of Medical Sciences/Peking Union Medical College, Beijing, China

## Abstract

Antiarrhythmic drugs (AADs) have a therapeutic effect on atrial fibrillation (AF) by regulating the function of ion channels. However, several adverse effects and high recurrence rates after drug withdrawal seriously affect patients' medication compliance and clinical prognosis. Thus, safer and more effective drugs are urgently needed. Active components extracted from natural products are potential choices for AF therapy. Natural products like Panax notoginseng (Burk.) F.H. Chen, Sophora flavescens Ait., Stephania tetrandra S. Moore., Pueraria lobata (Willd.) Ohwi var. thomsonii (Benth.) Vaniot der Maesen., and Coptis chinensis Franch. have a long history in the treatment of arrhythmia, myocardial infarction, stroke, and heart failure in China. Based on the classification of chemical structures, this article discussed the natural product components' therapeutic effects on atrial fibrillation by regulating ion channels, connexins, and expression of related genes, in order to provide a reference for development of therapeutic drugs for atrial fibrillation.

## 1. Introduction

**Figure 1:**
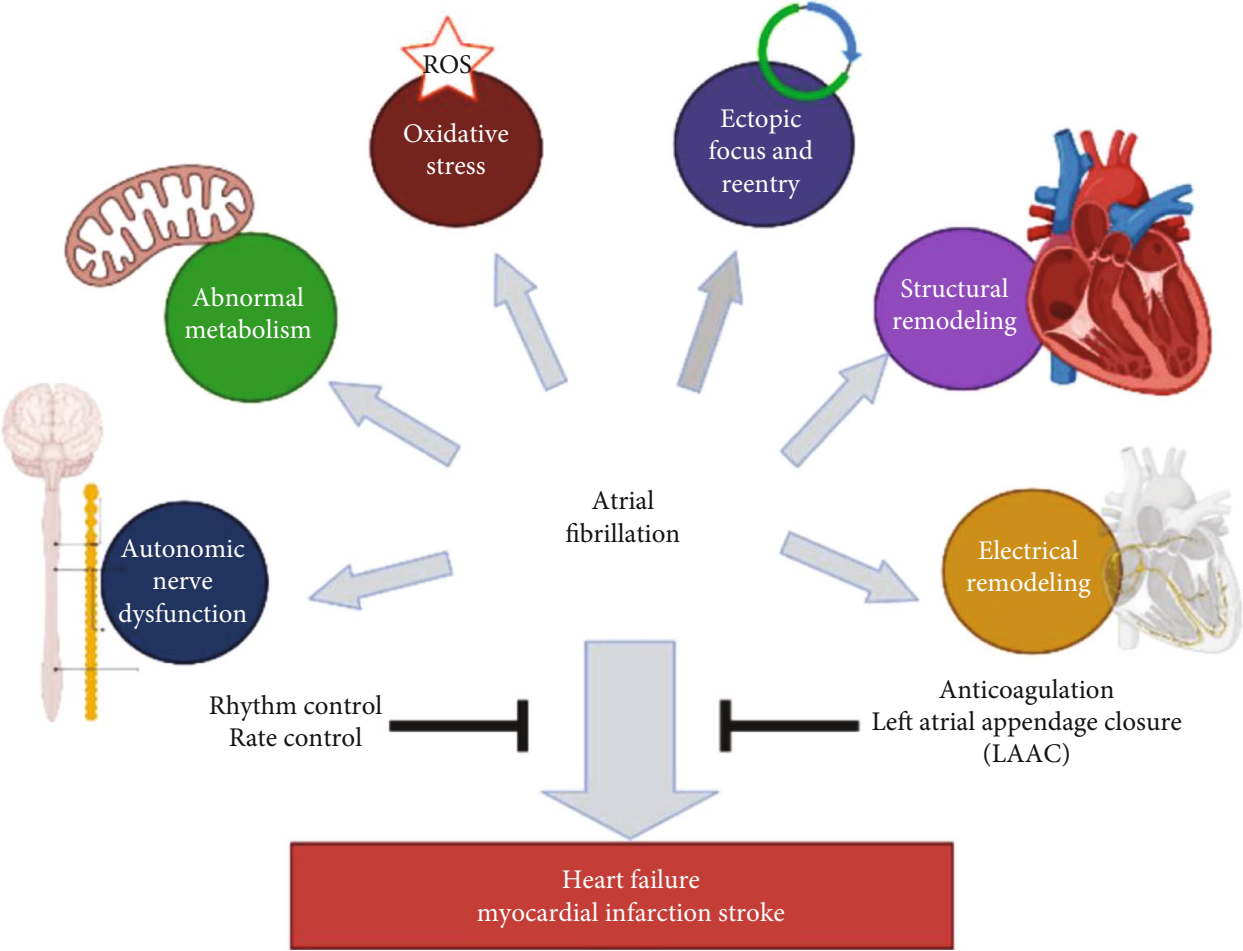
Pathophysiological changes, treatment, and complications of atrial fibrillation.

Atrial fibrillation (AF) is associated with a higher risk of stroke, heart failure with reduced ejection fraction, cardiomyopathy, acute coronary syndrome, and impaired quality of life. As shown in Figure 1, the pathophysiological changes of atrial fibrillation include oxidative stress, atrial structure, electrical remodeling, autonomic nerve dysfunction, metabolic abnormalities, ectopic activation, and reentry. AF treatment mainly includes rate and rhythm control therapy, anticoagulation, and left atrial appendage closure. Restoration and maintaining sinus rhythm have shown superiority in improving survival, quality of life, and ventricular function and reducing heart failure hospitalization. British National Institute for Health and Care Excellence (NICE) guideline [1], 2019 American Heart Association (AHA)/American College of Cardiology (ACC)/Heart Rhythm Society (HRS) AF guideline [2], and 2020 the European Association of Cardio-Thoracic Surgery (EACTS) [3] all recommended catheter ablation (mainly including radiofrequency ablation and cryoballoon ablation) for sinus rhythm recovery in patients with atrial fibrillation. However, after primary catheter ablation, the recurrence rate of AF/AF is high, especially in patients with severe structural cardiac remodeling, chronic kidney disease, and hyperthyroidism. Some patients have to receive more than one catheter ablation or be followed with electric cardioversion [4].

AADs have a therapeutic effect on atrial fibrillation by regulating potassium, calcium, sodium channels, or *β*1 receptors. However, adverse reactions can seriously affect the prognosis of patients with atrial fibrillation. Propafenone, sotalol, and ibutilide may give rise to severe ventricular arrhythmia. Amiodarone may cause interstitial lung disease, thyroid dysfunction, and nonalcoholic fatty liver disease. Natural products have unique advantages in antiarrhythmia, with few side effects and rarely inducing other arrhythmias. In recent years, it has become a hotspot to search for antiarrhythmia active ingredients from natural products. In this article, natural products with effects on more than two kinds of ion channels were included. By taking chemical structure as classification standard, natural products' antiarrhythmic effects on ion channels and target genes were discussed in detail to lay a foundation for the follow-up research and development of antiarrhythmic drugs.

## 2. Ion Channels and Connexins in the Pathogenesis of Atrial Fibrillation

As signal detectors, relayers, and amplifiers, ion channels regulate signal transduction and ion transport across cell membranes [5]. The abnormal function of ion channels increases vulnerablity and sustainability of AF [6]. Recurrence of atrial fibrillation can lead to a shorter effective refractory period of atrial myocytes, increased dispersion, and decreased or disappearance of frequency adaptability, thus promoting the deterioration of paroxysmal atrial fibrillation into persistent atrial fibrillation. Moreover, ion channel coding genes related to atrial fibrillation have also been discovered. In patients with atrial fibrillation, decreased expression of L-type Ca 2 + channel, ryanodine receptor (RyR2), potassium voltage-gated channel subfamily A member 5 (KCNA5), sarcoplasmic reticular Ca2+-ATPase (SERCA2), the beta-subunit MinK (KCNE1) and MIRP2 (KCNE3) [7], and increased expression of hyperpolarization-activated cation channel two associated with the pacemaker current I (f) (HCN2) were observed [8]. The left atrial diameter was negatively correlated with the expression of RyR2 and KChIP2 [7].

### 2.1. Potassium Channel

Four major kinds of potassium channels have been identified in cardiovascular myocytes, including inwardly rectifying (Kir) K+ channels, calcium-activated potassium channel (KCa), voltage-gated (VK), and two-pore domain potassium channels (K2P) [5]. (Kir) current (IK1) and acetylcholine-activated potassium current (IK, Ach) are involved in AF drivers formation (rotors and focal impulses) [9–11]. The increase of vagus nerve tension can enhance IKAch to shorten APD and stabilize the rotor related to reentry [12]. KCa channels can be divided into the big, small, and intermediate-conductance K+ channels [13]. KCa channels are also related to atrial structural and electrical remodeling in atrial fibrillation [14]. Transient outward current (Ito), similarly to the ultrarapid current (IKur), contributes to early repolarization [15]. By starting membrane depolarization, VK allows K+ efflux and regulates cardiac action potential duration. K2P- channels conduct outward K+ currents and modulate action potential repolarization [16].

### 2.2. Calcium Channel

L-type calcium current is the primary inward current in the action potential plateau phase, while T-type calcium current depolarizes current in phase 0 of action potential duration [17]. Generated and conducted from the sinoatrial node and atrioventricular node, L-type Ca2+ current was not only the primary inward current in atrial and ventricular action potential 2 phases [18]. Depolarization of cardiomyocytes can open the L-type calcium channel and the influx of Ca2+ and then trigger the release of Ca2+ from the sarcoplasmic reticulum. The process above is essential in the excitation-contraction coupling of cardiomyocytes [19].

Abnormal intracellular calcium (Ca2+) handling can trigger delayed after depolarization (DADs) and thus increase atrial ectopic activity. Intracellular calcium- (Ca2+-) calmodulin- (CaM-) calmodulin kinase (CaMK II) signal transduction pathway plays a central role in the regulation of intracellular calcium. Increased spontaneous sarcoplasmic reticulum (SR) Ca2+ release leads to ryanodine receptor (RyR2) dysregulation and Ca2+/calmodulin-dependent protein kinase II (CaMKII) hyperactivity. Exciting *β* adrenoceptors can enhance RyR2 receptor phosphorylation and promote ectopic activation associated with delayed depolarization [20]

### 2.3. Sodium Channel

The voltage-gated sodium channels contribute to the initiation and conduction of action potential [21, 22]. Prolonging sodium influx in the plateau phase can lead to early afterdepolarizations and ventricular tachycardia [23]. The sequential activation and inactivation of sodium channels prevent proarrhythmic events [24]. In recent years, late sodium current has been related to atrial fibrillation [25]. It promotes the occurrence of AF by increasing the dispersion of repolarization and leads to intracellular calcium overload.

### 2.4. Connexin

Cardiac connexins contribute to gap junctions intercellular electrical and molecular signaling communication [26]. There are three different connexins in the human heart, including Cx40, Cx43, and Cx45 [27, 28]. Connexin 45 is mainly expressed in sinus node (SA) and atrioventricular node (AV), while connexin 43 and connexin 40 are both expressed in atrial muscle [29]. Cx40 promoter polymorphisms that inhibit the expression of Cx40 are associated with the early onset of AF [30]. Somatic and germline mutations within the coding regions of the human Cx40 gene (GJA5) are also related to a higher risk of AF [31]. Atrial fibrillation leads to less connexin protein, less intercellular electrical coupling, changes in the electrical conductivity of the myocardium, the conduction velocity, and an aggravated degree of the auriculoventricular block.

### 2.5. Hyperpolarization-Activated Cyclic Nucleotide-Gated Cation Channel (HCN)

HCN2 and HCN4 are channels responsible for cardiac hyperpolarization-activated cation current (or “funny current”, If) [32, 33]. Their function decreases in the sinoatrial node and increases in the atrial and pulmonary vein in atrial fibrillation patients [34]. Activated by intracellular cAMP and membrane hyperpolarization, cardiac hyperpolarization-activated cation current contributes to diastolic depolarization of pacemaker cells [35].

## 3. Natural Products with Bioactivity in AF

### 3.1. Saponin

Saponins are glycosides with triterpenes or helical steranes as aglycones. Saponins are the main practical components of Panax notoginseng (Burkill) F. H. Chen ex C. H., Radix Polygalae, Platycodon grandiflorus, and Radix Bupleuri [36].

#### 3.1.1. Panax notoginseng Saponins (PNS)

Extracted from the roots of Panax notoginseng (Burk.) F.H. Chen, PNS has antiarrhythmia, antiplatelet [37], antishock [38], antioxidation [39], sedative [40], and antitumor [41] effects. Panax notoginseng saponins have been approved in the treatment of central retinal vein occlusion, sequelae of cerebrovascular disease, enophthalmos, and anterior chamber hemorrhage in China. By regulating potassium and calcium ion channels, PNS reduced the automaticity of cardiomyocytes, slowed down cardiac electrical conduction, prolonged action potential duration (APD) and effective refractory period (ERP), and prevented reentry agitation [42].

*(1) Potassium Channels*. PNS (150 mg/kg intraperitoneal injected once) significantly inhibited myocardial cell apoptosis induced by isoproterenol in atrial fibrillation model mice [43]. PNS significantly downregulated the expression of miR-499 in atrial tissues compared with the control group (*P* < 0.05). Small conductance calcium-activated potassium channel 3 (SK3) plays an essential role in the development of atrial fibrillation. Knocking out the SK3 gene could trigger atrial fibrillation. By downregulating, the expression of potassium calcium-activated channel subfamily N member 3(KCNN3) and SK3 and microRNA-499 (miR-499) affected the activity of the SK3 pathway and then triggered the occurrence of atrial fibrillation.

*(2) Calcium Channel*. CACNA1C gene encoded the L-type Ca2+ channel *α*1 subunit (Cav1.2) [44]. PNS (0.1, 0.6, 1, and 4 g/L) [45] inhibited L-type calcium channels (Cav1.2) and T-type calcium channels (Cav3.1) in Xenopus laevis oocytes in vitro. MicroRNA29 (miR29) targeted extracellular matrix proteins. It played an important role in the atrial fibrotic remodeling of AF and chronic heart failure patients [46]. By regulating the expression of the ATPase sarcoplasmic/endoplasmic reticulum Ca2+ receptor 2(ATP2A2) gene, miR-328 affected the intracellular Ca2+ concentration and the expression of calcineurin [47]. CACNA1C and CACNB1 that respectively encoded *α*1c- and *β*1 subunits of cardiac L-type Ca2+ channel were potential targets of miR-328 [48]. PNS [44] (150 mg/kg intraperitoneal injected once) significantly upregulated the expression of miR-29b and miR-328 (*P* < 0.05), thereby inhibiting isoproterenine-induced atrial fibrillation. In addition, PNS reduced the release of calcium in the sarcoplasmic reticulum, thus improving the apoptosis of myocardial cells caused by calcium overload [49].

In addition to regulating ion channels, the therapeutic effects of PNS on atrial fibrillation were also associated with anti-inflammation, antifibrosis, and antioxidative stress effects. After seven days of intraperitoneal injecting PNS (100 *μ*g/g), atrial fibrillation induced by ACh-CaCl2 mixed solution was significantly inhibited with effective refractory period prolonged, and the duration of atrial fibrillation shortened [50]. By activating the PI3K-AKT signaling pathway, the infiltration of inflammatory cells into cardiomyocytes and blood vessels and the deposition of collagen fibers around blood vessels were inhibited, with myocardial fibrosis improved in rats with atrial fibrillation [51].

In clinical trials, the therapeutic effect of PNS on atrial fibrillation has also been confirmed. Thirty-five patients with atrial fibrillation in the treatment group were given PNS (orally taking 100 mg, tid, for six months) combined with amiodarone (orally taking 200 mg, tid, for the first week, 200 mg bid for the second week, and continued with 200 mg once a day until six months), and 35 patients in the control group were given amiodarone (the same dosage as above, for six months) [52]. The AF recurrence rate in the treatment group was significantly lower than that of the control group (14.29% vs. 40%, *P* < 0.05). Left atrial diameter and ankyrin repeat expression in the treatment group were lower than those in the control group after six months of treatment. These results suggested that PNS inhibited atrial remodeling and ectopic pacing.

### 3.2. Alkaloids

Alkaloids are alkaline organic compounds containing nitrogen. They have alkaline properties and are widely distributed in advanced plants, especially in dicotyledonous plants.

#### 3.2.1. Berberine

Extracted from Coptis chinensis Franch. of Ranunculaceae and Mahonia fortunei (Lindl.) Fedde of berberis [53], berberine is a kind of isoquinoline alkaloid with antiarrhythmia [54], anti-inflammatory [55], antibacterial [56], hypoglycemia [57], vasodilation [58], and antitumor effects [59]. In terms of antiarrhythmia, berberine can prolong the action potential duration, and effective refractory period of myocardial cells [60] inhibits the occurrence of atrioventricular reentrant tachycardia by regulating potassium, calcium ion channels, and hyperpolarization-activated cyclic nucleotide-gated cation channel [61].

*(1) Potassium Channel*. Berberine could inhibit the rapid and slow activation of delayed rectifier outward potassium current in cardiomyocytes [62]. Berberine (3-100 *μ*mol/L) inhibited cromakalim-induced outward currents in isolated guinea-pig ventricular myocytes and ATP-sensitive K+ (KATP) channels [63]. Berberine (100 *μ*mol/L) had a blocking effect on inward rectifier potassium current (IK1) and outward delayed rectifier potassium current (IK) expressed in Xenopus oocytes. In vivo, berberine (10 and 20 mg/kg) inhibited the expression of KCNH2 in rat myocardium (*P* < 0.05) [64, 65].

*(2) Calcium Channel*. In vitro, berberine (10 and 30 *μ*mol/L) could not only inhibit the L-type and T-type calcium channel of guinea pig ventricular myocytes and inhibit extracellular Ca2+ influx but also reduce the delayed depolarizing induced by calcium overload [66, 67]. CPU86017 was a berberine derivative that could relieve heart failure by inhibiting calcium leakage, downregulating phosphatase, and exerting antioxidant activity [68]. Moreover, CPU86017 led to a regression of the transmural dispersion of repolarization and inhibition of RyR2 and SERCA2 [69].

*(3) Hyperpolarization-Activated Cyclic Nucleotide-Gated Cation Channel (HCN)*. Berberine (1-300 *μ*mol/L) inhibited the current of hyperpolarization-activated cyclic nucleotide-gated 4 (hHCN4) channel expressed in Xenopus laevis oocytes. Berberine decreased the rate of pacemaker firing and diastolic depolarization and changed the potential action parameters [70].

In clinical trials, Zheng et al. [71] conducted a retrospective cohort study of 88 patients with paroxysmal atrial fibrillation. Forty-five patients orally took berberine (the average dose of 1.3 g/day for one year), and 43 patients orally took amiodarone (0.2 g tid for the first week, 0.2 g bid for the second one, and 0.2 g for the following weeks, lasted for one year). There was no significant difference in the conversion rate and echocardiographic parameters between the berberine and amiodarone groups after 12 months of treatment. Echocardiographic parameters showed that the E/A ratio and left atrial diameter were significantly improved after 6 and 12 months of berberine treatment. However, in the amiodarone group, only E/A ratio got considerably enhanced.

#### 3.2.2. Tetrandrine (Tet)

Extracted from rhizomes of Trichosanthes Merr. Chun. of tetrandrine and roots of Stephania discolor, Stephania tetrandra, and Aristolochia heterophylla, tetrandrine is a kind of dibenzyl isoquinoline alkaloid [72] with antiarrhythmia [73], antihypertensive [74], anti-inflammatory [75], and antitumor [74] effects [76]. In terms of antiarrhythmia, by inhibiting calcium, potassium, and sodium channels, tetrandrine can slow down the heart rate, inhibit atrioventricular conduction, and prolong the effective refractory period of cardiomyocytes [77].

*(1) Potassium Channel*. Tetrandrine dosage-dependently inhibited delayed rectifier potassium current. The maximum effective concentration is 3 × 10^−5^ mol/L [18]. Tetrandrine had a bidirectional regulation effect on calcium-activated potassium channels. In vitro, tetrandrine (7.5 and 15 *μ*mol/L) increased the opening frequency and prolonged the opening time of calcium-activated potassium channels in rabbit cardiomyocytes. However, at the concentration of 30 *μ*mol/L, it significantly reduced the opening frequency and shortened the opening time of calcium-activated potassium channels.

*(2) Calcium Channel*. Tetrandrine could inhibit both L-type and T-type calcium channels in cardiomyocytes [78, 79]. Tetrandrine (6 *μ*mol/L) could reversibly block more than 50% of the intracellular Ca2+ current in rabbits' cardiomyocytes [80]. In isolated rats' cardiomyocytes, tetrandrine (100 micromol/L) reduced Ca2+ influx in the sarcolemma and inhibited Ca2+ uptake into the sarcoplasmic reticulum by inhibiting ATP2A2 [81]. Tet (50 mg/kg/d, intragastrically administrated for nine weeks) [82]) significantly inhibited calcium overload by reducing the density and the total number of dihydropyridine binding sites in the myocardium and vessels. Tet also improved left ventricular compliance and vascular endothelial function.

*(3) Sodium Channel*. Tet (10-30 *μ*mol/L) inhibited sodium current in single bullfrog cardiac cells [83]. Tet (40-120 *μ*mol/L) inhibited sodium current in cardiomyocytes from 12 patients with atrial fibrillation without affecting the density and properties of sodium channels [84]. The voltage-gated cardiac sodium channel contributed to action potential conduction [22]). Dysfunction of sodium channels increased susceptibility to atrial fibrillation.

#### 3.2.3. Matrine

Extracted from the dried root of Sophora flavescent Ait. and Euchresta japonica, matrine has antiarrhythmia [85], antibacterial [56], antipulmonary [86], hepatic fibrosis [87], and antitumor [88] effects. As a broad-spectrum antiarrhythmia drug, matrine can prolong action potential duration and effective refractory period, slow down heart rate, improve myocardial contractility, and inhibit ectopic rhythm and atrioventricular reentry agitation by inhibiting potassium, sodium, and calcium channels [89].

*(1) Potassium Channel*. The HERG gene (human ether-a-go-go-related gene), also known as KCNH2, was responsible for encoding Kv11.1 protein [90]. This potassium ion channel activates the cardiac delayed rapid-rectifying potassium current (IKr), which was responsible for action potential platform and 3-phase repolarization [91, 92]. IKr, IKs, and IK1 were repolarization reserve currents [93].

Matrine had a bidirectional regulation effect on the HERG potassium channel [94]. In vitro, a low concentration of marine (1 *μ*mol/L) promoted the expression of HERG in rats' cardiomyocytes. In contrast, a high concentration of matrine (100 *μ*mol/L) inhibited the expression of HERG, prolonged the action potential duration and effective refractory period (ERP) of ventricular myocytes, gradually slowed down the frequency of spontaneous discharge, and reduced the incidence of ectopic rhythm [95].

M3 receptor-mediated K+ current (IKM3) has been found to be a new target for the treatment of atrial fibrillation in recent years. Pretreatment of matrine (15, 30, and 45 mg/kg intravenously administrated once a day for 15 days) significantly reduced AF incidence rate and duration time in a dose-dependent manner. Matrine inhibited atrial repolarization by inhibiting IKM3 current, prolonged the effective refractory period, and made the effective refractory period in different parts of the myocardium tend to be the same, thus blocking the atrioventricular reentry excitation [96]. Expression of the M3 receptor was decreased, and Cav1.2 expression was upregulated on the atrial membrane [94]

Potassium inward rectifier channel Kir2 (encoded byKCNJ2) was responsible for terminal cardiac repolarization and resting membrane stability [97]. “Loss-of-function” or “Gain-of-function” mutations of KCNJ2 gave rise to atrial fibrillation. Kv 2.1 (encoded by KCNB1) could be downregulated in myocardial infarction patients and lead to electrical instability of the post-MI heart [98]. Matrine (50, 100, and 200 mg/kg, intragastrically administrated for seven days) [99] upregulated the expression of KCNB1 (encoding Kv 2.1) and KCNJ2 (encoding Kir2.1) in myocardial tissues of rats with myocardial infarction and prevented the occurrence of arrhythmia after myocardial infarction.

*(2) Calcium Channel*. Matrine (15, 30, and 45 mg/kg intravenously administrated once a day for 15 days) upregulated Cav1.2 expression on atrial membrane. It promoted the increase of L-type calcium current and the recovery of calcium-induced calcium release (CICR), which ultimately improved myocardial contractility and cardiac function and prevented heart failure in rats with AF [100]. Moreover, matrine (100 mg/kg/d, intragastrically administrated for four weeks) improved atrial fibrosis and reduced the susceptibility of AF in rats with myocardial infarction by inhibiting the proliferation, migration, and differentiation of cardiac fibroblasts [101].

*(3) Sodium Channel*. Voltage and concentration-dependently, matrine inhibited sodium channels. In vitro, matrine (10, 50, and 100 *μ*mol/L) inhibited sodium current in rat's ventricular myocytes and reduced the action potential amplitude (APA) [102, 103].

#### 3.2.4. Dauricine (Dau)

Dauricine is a kind of bibienyl isoquinoline alkaloid extracted from the roots of Menispermum dauricum DC., with antiarrhythmia [104], anti-inflammatory [105], antitumor [106], and anticoagulation [107] effects. In terms of antiarrhythmia, dauricine reduced Ca2+ and Na+ influx and K+ outflow. Dauricine (200 *μ*g/mL) [108] prolonged the atrial effective refractory period and action potential duration and significantly inhibited Na+, K+, and Ca2+ current in myocardial tissues.

*(1) Potassium Channel*. In guinea pig ventricular myocytes, dauricine (1, 3, 10, 30, and100 *μ*mol/L) inhibited the rapidly and slowly activating component of the delayed rectifier potassium current and the inward rectifier potassium current [85]. Dau inhibited both active and inactive states of HERG channels [109]. Unlike quinidine and dofetilide, dauricine did not affect the deactivation process of Ikr and Iks and was not likely to cause torsade de pointes ventricular tachycardia [110].

*(2) Calcium Channel*. Dauricine was an L-type calcium channel blocker that reduced intracellular Ca2+ concentration by inhibiting Ca2+-ATPase activity and sarcoplasmic reticulum calcium uptake. In rabbit papillary cardiomyocytes, dauricine (30 *μ*mol/L) inhibited early depolarization by inhibiting L-type calcium channels [111, 112]. Dauricine's inhibiting effects of Ca2+ channels were also associated with activated Na+-K+-ATPase and Ca2+-Mg2+-ATPase [113].

*(3) Connexin*. Decreased expression or function of connexin 40 protein promoted the aggravation of paroxysmal atrial fibrillation into persistent atrial fibrillation [114]. Dauricine (intravenous injecting 5 mg/kg, 30 min before rapid atrial pacing) inhibited the degradation of Cx40 and the damage of atrial myocytes caused by rapid atrial pacing [115].

#### 3.2.5. Guanfu Base A (GFA)

Extracted from the roots of Aconitum coreanum (Levl.) Rapaics of Ranunculaceae, GFA has antiarrhythmia [116], anti-inflammatory [117], and antioxidation [118] effects. At present, it has been approved in the treatment of supraventricular tachycardia in China. In terms of antiarrhythmia, GFA is a kind of multichannel blocker, which mainly blocks sodium channels. GFA can not only restrain action potential amplitude (APA) and Vmax and prolong action potential duration and effective refractory period but also change unidirectional conduction block into bidirectional conduction block and reduce the occurrence of premature contraction and atrioventricular reentry [119, 120].

*(1) Potassium Channel*. GFA mainly inhibited slow-activated delayed rectifier potassium current (Iks), with little influence on rapid-activated delayed rectifier potassium current (Ikr) [121]. Therefore, it was less likely to cause other arrhythmias [122]. In a frequency-dependent manner, GFA (100, 400, 1000, and 2500 *μ*mol/L) inhibited potassium currents by binding to the S6 region of the HERG channel without affecting the synthesis of HERG proteins. It inhibited the expression of HERG proteins at high concentrations [123]. GFA did not affect the inward potassium current channel and transient outward potassium current channel, so it would not lead to early repolarization [124].

*(2) Calcium Channel*. By reducing Ca2+ influx, GFA reduced the depolarization rate and average repolarization rate of cardiomyocytes [125]. GFA mainly acted on the inactive state of the L-type calcium channel, which prolonged recovery time from the inactivation state. GFA also had a certain effect on the calcium channel in the inactive state [126]. GFA (25, 125, 250, and 1000 *μ*mol/L) blocked L-type calcium channel in rats' ventricular myocytes in a concentration-dependent manner.

*(3) Sodium Channel*. By inhibiting the sodium channel, GFA reduced the heterotopic automaticity of atrial and ventricular cells [117]. GFA not only reduced the occurrence of reentry by slowing down the atrioventricular bypass conduction but also prolonged the action potential duration and effective refractory period. In vitro, GFA (500 *μ*mol/L) reduced the depolarization rate and average repolarization rate of rabbits' sinus node cells [127].

In addition, GFA could inhibit the late sodium channel, which was considered as a potential drug target of AF [128]. Late sodium current increased intracellular sodium and calcium loading [98] and enhanced susceptibility to atrial fibrillation [104], heart failure [129], and hypoxia [130]. Mutation in SCN5A (sodium voltage-gated channel alpha subunit 5) gene increased late sodium current and gave rise to malignant arrhythmia with pleomorphic ventricular tachycardia and torsade de pointes (TdP). By inhibiting the expression of the SCN5A, GFA (100 *μ*mol/L) shortened the recovery time of action potential and restrained the triggered activity caused by early after depolarization and delayed after depolarization [131].

*(4) Connexin*. GFA (6 and 12 mg/kg, intragastrically administrated for four days) shortened the duration of atrial fibrillation in calcium chlorine-acetylcholine model rats, prolonged the effective refractory period, reduced the expression of NADPH oxidase-related subunits, and promoted the communication junction protein expression (connexin 40). In this way, it inhibited atrial electrical remodeling caused by atrial fibrillation and increased the success rate of conversion to sinus rhythm.

In a clinical trial, 41 patients with atrioventricular reentrant tachycardia in the treatment group were intravenously injected GFA (200 mg, if the first dose is ineffective, the second dose may be given after 15 minutes), and 41 patients in the control group intravenously were injected propafenone hydrochloride (70 mg, intravenously injected, if the first dose is ineffective, the second dose may be given after 30 minutes) [132]. The results showed that the effective rate of GFA hydrochloride in the treatment of atrioventricular reentrant tachycardia was higher than that of propafenone hydrochloride (87.8% vs. 68.3%, *P* < 0.05). The recovery time of collateral retrograde transmission in the GFA group was longer than that in the propafenone group (36.6 ± 9.7 vs. 19.2 ± 7.3 min, *P* < 0.05).

#### 3.2.6. Neferine

Extracted from the seeds of lotus of Nymphaeaceae, neferine has antiarrhythmia, antihypertensive [133], antitumor [134], antiapoptotic, antioxidative, [135], anti-ischemic [136], antiallergic, and anti-inflammatory effects [137]. In terms of arrhythmia, by reducing the Na+, Ca2+ ion influx, and K+ outflow, nephrine can effectively prolong the Q-T interphase and slow down heart rate [138].

*(1) Potassium Channel*. By blocking the HERG potassium channel, neferine (10 and 30 *μ*mol/L) inhibited potassium outflow during repolarization, prolonged the action potential duration, and effective refractory period [116, 140]. In vitro, neferine (10 and 100 *μ*mol/L) reduced the peak value of potassium current in rabbit ventricular myocytes by 56.96% and 73.61%, respectively [65].

*(2) Calcium Channel*. Concentration dependently, neferine blocked the L-type calcium channel and had a synergistic effect with verapamil. In vitro, neferine reduced the peak calcium current of rabbit ventricular myocytes by 17.46% and 51.06% at concentrations of 10 and 100 *μ*mol/L (Zuo et al., 2021 [140]). Neferine inhibited intracellular calcium influx induced by adenosine diphosphate (ADP) by inhibiting Ca2+ influx and internal Ca2+ discharge [141].

*(3) Sodium Channel*. Neferine (30 *μ*mol/L) inhibited activation of Nav1.5 currents in a frequency-dependent manner [38] in HEK293 cells. Neferine (40 *μ*mol/L) reduced the action potential amplitude (APA), maximum rising rate (Vmax), and maximum rate of depolarization in rats' cardiomyocytes [142]. Neferine significantly inhibited the function of the sinoatrial node and the conduction of electrical current between atrial and ventricular cells [110].

### 3.3. Quinones

#### 3.3.1. Tanshinone IIA

Extracted from the root of Salvia miltiorrhiza Salvia miltiorrhiza Bunge., tanshinone?A has anti-arrhythmic [143], anticoagulant, anti-ischemic [144], anti-neoplastic [145], and anti-inflammatory [146] effects. By inhibiting potassium and calcium ion channels, tanshinone IIA can prolong the effective refractory period and action potential duration, inhibit cardiac repolarization, and increase the threshold of ventricular fibrillation [147].

*(1) Potassium Channel*. The KCNJ2 gene that encoded Kir2.1 and Kir2.2 proteins of inward rectifier potassium channels [121] was associated with the pathogenesis of atrial fibrillation [148]. KCNQ1 gene-encoded Kv7.1 protein of Iks, while KCNE1 encoded Mink protein of Iks channel. In myocytes of AF rats, tanshinone IIA upregulated the expression of KCNJ2, downregulated the expression of KCNQ1 and KCNE1, and inhibited myocardial cell potassium outflows and repolarization of cardiomyocytes [129, 149]. In vivo, tanshinone IIA (2 mg/kg) significantly prolonged the rabbit ventricular relative refractory period, effective refractory period [150]. Because of little impact on the Ikr channel, tanshinone IIA was less arrhythmogenic than sotalol.

*(2) Calcium Channel*. Tanshinone IIA (32 mg/kg, intragastrically administrated for 14 days) significantly upregulated the expression of the CACNA1C gene in atrial tissue of AF rats and improved atrial electrical remodeling and calcium overload [41]. Tanshinone IIA reduced the expression of microRNA-1 through the p38 mitogen-activated protein kinase pathway [129, 151]. In addition, tanshinone IIA inhibited collagen secretion induced by AngII and the synthesis rate of atrial fibroblasts by means of inhibiting the TSP-1/TGF-*β*1 pathway [152]. The differentiation of atrial fibroblasts into myofibroblasts plays an important role in atrial fibrosis. As a water-soluble derivative of tanshinone IIA, sodium tanshinone IIA sulfonate prevented atrial fibrosis by inhibiting oxidative stress and TGF-*β* activation in the AngII-1 signaling pathway [153].

In China, tanshinone IIA has been widely used in the clinical treatment of myocardial infarction complicated by atrial fibrillation. Wang and Xie [154] conducted a meta-analysis that included ten clinical studies (involving 1088 patients). The results showed that success rate of conversion into sinus rhythm (OR = 3.10, 95%CI = 2.16-4.44, *P* < 0.05), the recurrence rate of atrial fibrillation (OR = 0.32, 95%CI = 0.22-0.48, *P* < 0.05), the incidence rate of heart failure (OR = 0.23, 95%CI-0.15-0.35, *P* < 0.05), mortality (OR = 0.23, 95%CI = 0.15-0.35, *P* < 0.05), and adverse reaction rate (OR = 0.24, 95%CI = 0.12-0.49, *P* < 0.05) in the treatment group (Tanshinone IIA sulfonate combined with amiodarone) were better than those in the control group (only amiodarone).

### 3.4. Polyphenols

Polyphenols are secondary metabolites with polyatomic phenol structures that are widely found in the skin, roots, leaves, and fruits of medicinal plants like Polygonum cuspidatum Sieb.et Zucc., Syringa oblata Lindl., and Paeonia suffruticosa Andr. Among them, resveratrol, puerarin, and acacetin have been found with good antiarrhythmic effects.

#### 3.4.1. Resveratrol

Extracted from dry roots of Polygonum cuspidatum Sieb.et Zucc. of Polygonaceae, resveratrol has antiarrhythmia [155], antioxidation [156], antitumor [157], anti-ischemic [158], and antiplatelet [159] effects. In terms of antiarrhythmic, resveratrol can slow down the heart rate, prolong the effective refractory period of cardiomyocytes, and inhibit the occurrence of early and delayed after depolarization [158], by regulating potassium, calcium, and sodium ion channels [160].

*(1) Potassium Channel*. Resveratrol prolonged action potential duration and effective refractory period by inhibiting the expression of the HERG gene, as well as rapid and slow activation of delayed rectifying potassium ion current [161]. In vitro, resveratrol (50, 100, and 500 *μ*M) slowed down guinea pigs' heart rate and inhibited myocardial contractility in a dosage-dependent manner by regulating ATP-sensitive potassium channels, transient outward potassium current, calcium-activated potassium channels, and inward rectifying potassium channels [162].

Resveratrol enhanced Kv2.1 potassium current in H9C2-rat cardiomyocytes in a time- and concentration-dependent manner. The median maximum effective concentration was 14.02 *μ*mol/L [163].

*(2) Calcium Channel*. Resveratrol (1, 50, and 100 *μ*mol/L) inhibited the L-type calcium channel, reduced the intracellular calcium influx, and prolonged the effective refractory period in guinea pig ventricular myocytes [164, 165]. Resveratrol not only inhibited the occurrence of early and delayed after depolarization (EAD and DAD) but also slowed down the atrioventricular conduction and inhibited atrioventricular node reentry excitement. In vitro, resveratrol protected guinea pigs' ventricular myocytes from oxidative stress-induced arrhythmias and calcium overload, by means of inhibiting L-type calcium channel, reducing the production of oxygen free radicals in cardiomyocytes, and preventing the activation of calmodulin-activated protein kinase II (CaMK II) [155].

*(3) Sodium Channel*. Concentration-dependently, resveratrol (10, 30, and 100 *μ*mol/L) inhibited the late sodium current and the reverse type sodium-calcium exchangers of ventricular myocyte of guinea pig [166]. At the concentration of 100 *μ*mol/L, resveratrol could inhibit 52.7 ± 10.2% of sodium current [167]. Resveratrol (10, 20, 40, and 80 *μ*M) inhibited ischemic arrhythmias by inhibiting the H2O2-induced late sodium current and the reverse sodium-calcium exchange current (INCX) [168].

*(4) Connexin 43*. Resveratrol could regulate the expression of the GJA gene and myocardial connexin 43 (Cx43), which play a therapeutic role in idiopathic atrial fibrillation [169]. Resveratrol (1 mL/kg, intravenously administered) upregulated the expression and activity of Cx43 in male SD rats by activating the PI3K/Akt signaling pathway, thus preventing myocardial ischemia-reperfusion arrhythmia [170]. Resveratrol (2.5 mg/kg/d, intragastrically administrated for seven days) inhibited atrial remodeling and reduced AF by increasing activity of deacetylase 1 (SIRT1) [171].

*(5) HCN Channel*. Resveratrol inhibited hyperpolarization-activated cyclic nucleotide-gated cation channel 4 (HCN4) (IC50 value = 83.75 *μ*mol/L) [172]. Resveratrol inhibited Kv1.5 current and IKAch [173] frequency-dependently, with IC₅₀ of 0.36 and 1.9 *μ*mol/L [173].

#### 3.4.2. Puerarin

Extracted from the dry root of Pueraria puerariae, puerarin is a kind of flavonoid compound with antiarrhythmia [175], anti-ischemic [176], antihypertensive [177], hypolipidemic [178], hypoglycemic [179], coronary vasodilation [180], and anti-inflammatory [181] effects. In China, puerarin (Yufeng Ningxin Dropping Pill) has been approved for the clinical treatment of hypertension, coronary heart disease, angina pectoris, and neuropathic headache. In terms of antiarrhythmia, puerarin can slow down the heart rate, inhibit the cardiomyocyte automaticity, prolong the effective refractory period, and action potential duration [182, 183].

*(1) Potassium Channel*. Puerarin (0.01, 0.1, and 1 mmol/L) prolonged the duration of the action potential by inhibiting IKs in rat ventricular myocytes [184]. Zhang et al. [185] found that puerarin was a novel antagonist towards inward rectifier potassium channel (IK1). Puerarin (1.2 mmol/L) significantly inhibited the IK1 current in rat ventricular cells. In the transfected HEK293 cells, puerarin significantly inhibited inward rectifying K+ channels in a dose-dependent manner and had a more significant effect on Kir2.1 and Kir2.3. Puerarin significantly inhibited Kv7.1 and IKs in micromolar concentrations [186]. Moreover, by regulating the calcium-activated potassium channel and activating protein kinase C, puerarin (0.24 mmol/L) protected rats' ventricular myocytes against ischemia and reperfusion injury [187].

*(2) Calcium Channel*. Puerarin (2.4 mmol/L) inhibited the L-type calcium channel in rats' ventricular myocytes in a time-dependent manner [188]. Puerarin (100 mg/kg, iv) [189] significantly inhibited the arrhythmias (including ventricular premature contraction, ventricular tachycardia, and ventricular fibrillation) induced by chloroform (2 mL inhaled by mice) or aconitine (40 mg/L intravenous injected to SD rats). Puerarin ([190], 100 mg/kg intraperitoneally injected for ten days) inhibited isoproterenol-induced cardiac hypertrophy and reduced intracellular calcium concentration.

*(3) Sodium Channel*. M. Puerarin inhibited sodium channels in a dose-dependent manner with IC (50) = 349 *μ*mol/L [191]. Wang conducted a prospective cohort study involving 87 patients with persistent atrial fibrillation. Forty-three patients in the control group received amiodarone (0.2 g tid for one week, 0.2 g, bid for one week, and 0.2 g QD for the last week); 44 patients in the combination group received puerarin (40 mL + 250 mL 0.9% NaCl, ivgtt, for 21 days) based on the control group. The results showed that the average time of restoring sinus rhythm in the combination group was significantly shorter than that of the control group (7.5 d vs. 10.2 d, *P* < 0.01), and the success rate of restoring sinus rhythm was significantly higher than that of the control group (77.3% vs. 60.5%, *P* < 0.01) [192].

#### 3.4.3. Acacetin

Acacetin is a kind of flavonoid extracted from Saussurea involucrata (Kar. et Kir.) Sch.-Bip., with antiarrhythmic [193], antitumor [194, 195], anti-inflammatory [196], antifibrosis [197], and antioxidation [198]. In terms of antiarrhythmia, acacetin is a kind of potassium channel inhibitor without effect on the Na(+) and L-type Ca (2+) channels. Acacetin (3, 6, and 12 mg/kg, intravenously administrated for seven days) terminated experimental AF of beagle dogs without prolonging QTc interval [199].

*(1) Potassium Channel*. Acacetin (5–10 *μ*M) reduced Ito density, action potential notch, and J wave area in electrocardiograph [200]. Encoded by KCNA5, ultrarapid delayed rectifier potassium channel (Kv1.5) was only expressed in human atrial myocytes. Inhibiting the Kv1.5 current could prolong atrial action potential duration and increase the refractory period of the fibrillating atrium [14]. Acacetin (IC50 = 3.2 and 9.2 *μ*mol/L, respectively) inhibited ultrarapid delayed rectifier potassium current I (Kur) and the transient outward K+ current and IKACh in human atrial myocytes [201], with little potency in inhibiting IKr and IKs. Other flavonoids, including hesperetin [14], myricetin [202], and quercetin [203], could also inhibit I (Kur).

Blocking the K+ channel could enhance the AF-selectivity of INa blockade. As a K+-current blocker, acacetin combined with INa blocker showed synergistic antiarrhythmic benefits without significant alterations of ventricular repolarization and QT intervals.

Acacetin showed a weak inhibition in the hERG and KCNQ1/KCNE1 channels [193] in rabbit hearts. In cardiomyocytes of anesthetized dogs, acacetin (2.5, 5, and 10 mg/kg) prevented AF induction [193]. Chen et al. [204] found that acacetin inhibited small conductance Ca2+-activated K+ channel (SKCa) currents with IC50 of 12.4 *μ*M for SKCa1, 10.8 *μ*M for SKCa2, and 11.6 *μ*M for SKCa3.

### 3.5. Organic Acid

Organic acids are organic compounds with acidity that are widely distributed in leaves, roots, and fruits of Chinese Materia Medica like Glycyrrhiza uralensis Fisch.

#### 3.5.1. Glycyrrhizic Acid (GA)

Extracted from the dried root and rhizome of Glycyrrhiza uralensis Fisch., Glycyrrhiza inflata Bat., or Glycyrrhiza glabra L., glycyrrhizic acid has antiarrhythmia [205], antioxidant, anti-inflammatory [206], antiviral [181], and antitumor (Zuo et al., 2021) effects [207]. In terms of arrhythmia, glycyrrhizic acid can block sodium and calcium ion channels to inhibit the automaticity of pacemaker cells, slow down the conduction speed, prolong the action potential duration, and effective refractory period [208].

*(1) Calcium Channel*. Glycyrrhizic acid can act on the CACNA1C gene to inhibit the L-type Ca2+ channel current in cardiomyocytes. Therefore, it can hinder sinus node pacing early and delayed depolarizing triggering activities [205].

Adrenergic receptors of the heart stimulated by epinephrine and norepinephrine can promote the release of cAMP, phosphoinositide, and the second messenger signaling cascade [209]. In vitro, ten *μ*M GA can inhibit the cAMP levels of CHO cells transfected with *β*2-AR or *β*3-AR, suggesting its' selective antagonistic capability against *β*2-AR and *β*3-AR [205].

*(2) Sodium Channel*. Glycyrrhizic acid can inhibit sodium ion channels in cardiomyocytes in a concentration-dependent manner [210]. Glycyrrhizic acid can inhibit Na + influx of cardiac myocytes during depolarization, reduce action potential amplitude and maximum rise rate of the action potential, slow down conduction velocity, and reduce Na + influx in phase 4 of action potential duration in ectopic pacemaker cells, thus decreasing the excitability of ectopic pacers [211].

### 3.6. Terpenoids

Widely existing in natural products, terpenoids are compounds and derivatives derived from methyl glutaric acid and whose molecular skeleton takes isoprene unit as the basic structural unit.

#### 3.6.1. Artemisinin

Extracted from Artemisia annua L., artemisinin has antimalarial [212], antitumor [213], anti-inflammatory [214], antiviral [215], antibacterial [216], and antifibrotic [217] effects. Artemisinin (5, 10, and 20 mg/kg, intraperitoneally injected for once) inhibited arrhythmia induced by barium chloride by regulating sympathetic tone. Artemisinin was superior to amiodarone in prolonging QT interval (4.0 mg/kg, intraperitoneally injected), and there were fewer adverse reactions [180].

*(1) Potassium Channel*. Artemisinin (5 and 50 *μ*mol/L) inhibited the inward rectifier potassium channel (IK1) of African frog [218] and inhibited IK1, It0, and delayed activation rectifier potassium current of dogs' cardiomyocytes [219].

*(2) Calcium Channel*. Artemisinin (4, 2, and 1 mg intragastrically administrated for 28 days) upregulated Cav1.2 calcium channel expression level, downregulated the expression level of calmodulin and calmodulin-dependent protein kinase II (CaMKII), and inhibited the level of phosphorylated ryanodine receptor 2 [220].

*(3) Connexin 43*. Cx43 remodeling was associated with action potential duration dispersion and reducing conduction [221]. Artemisinin increased Cx43 expression by inhibiting TNF-*α* and attenuating sympathetic tone [222].

*(4) Hyperpolarization-Activated Cyclic Nucleotide-Gated Cation Channel (HCN)*. HCN mainly had the following characteristics: activated by hyperpolarization, sodium-potassium ion mixed channel, double regulated by voltage, and cyclic nucleotide [223]. HCN channel was the primary determinant of phase 4 automatic depolarization of autorhythmic cells and improved the autonomy of autorhythmic cells. Artemisinin (75 mg/kg, intragastrically administrated, three times a day for four weeks) inhibited the pacing current of sinoatrial node in rabbits with heart failure, reduced the expression of HCN channel, and thus reduced the heart rate [224].

## 4. Summary

**Figure 2:**
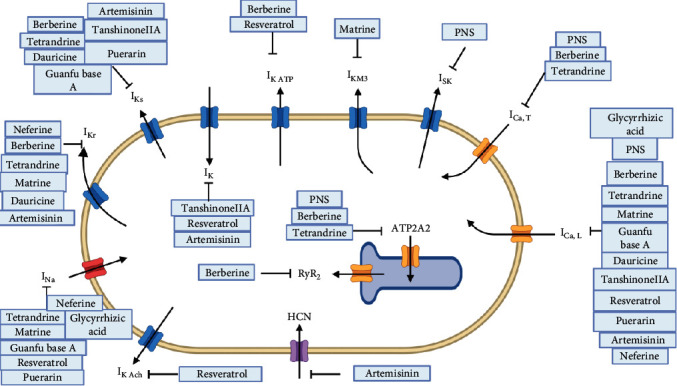
Natural products' effects on potassium, sodium, and calcium channels.

Based on the classification of chemistry structure, the effects of saponins, alkaloids, polyphenols' effects on potassium, sodium, and calcium channels of cardiomyocytes are summarized in this article (see Table 1 and Figure 2). In addition, the natural products' regulatory effects exerted on the expression level and function of genes responsible for encoding ion channel protein are also summarized (see Table 2).

However, most of the studies above are limited to animal or cell experiments, which only preliminarily demonstrate their therapeutic effect on atrial fibrillation. In the future, studies on pharmacokinetics/pharmacodynamics and mechanisms as well as clinical trials of natural products with antiarrhythmic activity should be performed. We will continue to track relevant reports to promote the development of new AADs.

## 5. Conclusion

Potassium, calcium, sodium channel, connexins, and HCN channel are involved in the pathogenesis of atrial fibrillation. In terms of potassium channels, predominantly expressed in atria, ultrarapidly delayed rectifier potassium channel (Kur) and small conductance calcium-activated potassium channel (SKCa) can be considered to be atrial-selective targets for developing anti-AF drugs. Kur can enhance spiral-wave reentry. SKCa can prolong atrial repolarization in the pulmonary vein and inhibit the maintenance of atrial fibrillation. Ectopic rhythm caused by EAD, DAD, and calcium overload can be inhibited by blocking L-type calcium channels or late sodium channels. Increasing the expression or function of connexins can inhibit reentry and heterogeneous conduction. HCN channel is a promising channel in heart rate control therapy by inhibiting diastolic depolarization.

Tanshinone IIA and Guanfu A have been approved in the clinical treatment of arrhythmia in China. The advantage of tanshinone IIA is inhibiting structural and electrical remodeling. The benefit of Guanfu A is inhibiting triggered activity and atrioventricular reentry. Although PNS, berberine, and puerarin have been found effective in clinical trials of paroxysmal atrial fibrillation, their efficacy still needs to be confirmed by well-designed, randomized, double-blind controlled trials.

## Figures and Tables

**Table 1:** The effects of active components of antiarrhythmic natural products exerted on ion channels.

Origin	Names	Pharmacological action	Chemical structure	Potassium channel	Calcium channels	Sodium channel	Connexin	HCN
*Panax notoginseng* (*Burk*.) *F.H. Chen*	Panax notoginseng saponins	Antiarrhythmia [42], antiplatelet [37], antishock [39], antioxidation [225], sedative [40], and antitumor [41]	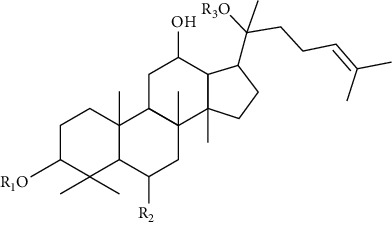	Small conductance calcium activates potassium channel, potassium calcium-activated channel [43]	L-type and T-type Ca2+ channel [44] [50]			
*Coptis chinensis Franch.*, *Phellodendron chinense Schneid*, *Mahonia bealei* (*Fort*.) *Carr*	Berberine	Antiarrhythmia [54], anti-inflammatory [55], antibacterial [56], hypoglycemia [57], vasodilation [58], antitumor effects [59].	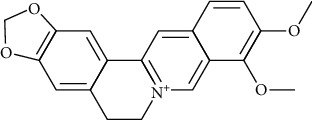	Delayed rectifier outward potassium channel, ATP-sensitive K+ (KATP) channels, inward rectifier potassium channel ([64, 65])	L-type and T-type calcium channel, ryanodine receptor, sarcoplasmic/endoplasmic reticulum calcium ATPase ([66]; [67]) [68]			hHCN4 [70]
*Stephania tetrandra S. Moore.*	Tetrandrine	Antiarrhythmia [73], antihypertensive [74], anti-inflammatory [75], and antitumor [74]	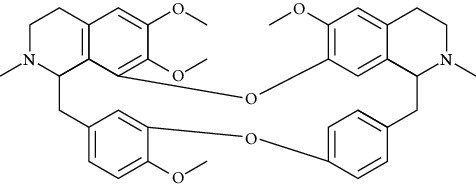	Delayed rectifier potassium channel, calcium-activated potassium channel [18]	L-type and T-type calcium channels, sarcoplasmic/endoplasmic reticulum calcium ATPase [80] [82]	Voltage-gated cardiac sodium channel [22] [84]		
*Sophora alopecuroides L*., *Sophora flavescens Alt*.	Matrine	Antiarrhythmia [85], antibacterial [226], antipulmonary [86], and hepatic fibrosis [87], and antitumor [88]	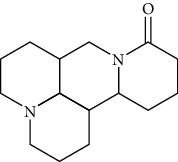	Delayed rectifier potassium channel, M3 receptor-mediated K+ channel [96] [94], potassium inward rectifier channel [98] [99]	L-type calcium channels [100] [101]	Voltage-gated cardiac sodium channel ([103]; [102])		
*Menispermum dauricum DC*.	Dauricine	Antiarrhythmia [104], anti-inflammatory [105], antitumor [106], anticoagulation [107]	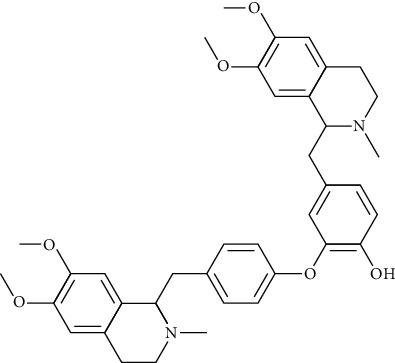	Delayed rectifier potassium channel, inward rectifier potassium channel [94] [95] [96]	L-type calcium channels [101] [100]		Cx40 ([27]; [28]) [29] [30] [31] [115]	
*Aconitum coreanum* (*Levl*.) *Rapaics*	Guanfu base A	Antiarrhythmia [116], anti-inflammatory [117], and antioxidation [118]	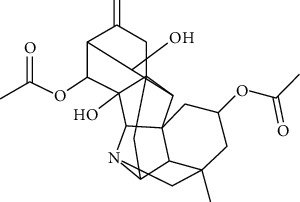	Delayed rectifier potassium channel [121] [122] [124]	L-type calcium [125] [126]	Voltage-gated cardiac sodium channel [117] [127]	Cx40 [132]	
*Lotus of Nymphaeaceae*	Neferine	Antiarrhythmia, antihypertensive [133], antitumor [134], antiapoptotic and antioxidative [135], anti-ischemic [136], antiallergic, and anti-inflammatory effects[137]	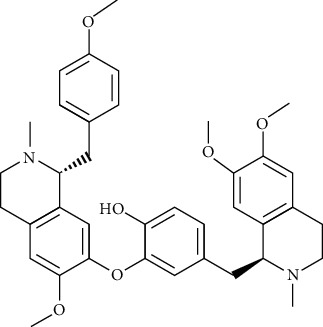	HERG potassium channel ([116]; [140])	L-type calcium channel (Zuo et al., 2021) [141]	Voltage-gated cardiac sodium channel [38] [142] [110]		
*Salvia miltiorrhiza Bge*.	Tanshinone IIA	Antiarrhythmic [143], anticoagulation, anti-ischemia [144], antitumor [145], and anti-inflammation [146]	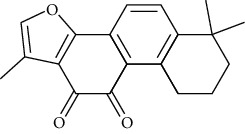	Delayed rectifier potassium channel ([129]; [149]), inward rectifier potassium channel [227] [148]	type calcium channel [41]	Voltage-gated cardiac sodium channel [152]		
*Vitis vinifera L*., *Punica granatum L*., *Vaccinium spp*., *Vaccinium macrocarpon*	Resveratrol	Antiarrhythmia [155], antioxidation [156], antitumor [157], antimyocardial ischemia [158], and antiplatelet [159]	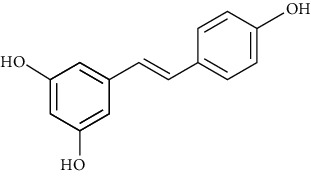	Delayed rectifier potassium channel [161], ATP-sensitive potassium channels, transient outward potassium channel, calcium-activated potassium channel, and inward rectifying potassium channel [163]	L-type calcium ([164]; [165]) [155]	Late sodium channel and reverse type sodium-calcium exchangers [166]	Cx43 [169] [170]	Hyperpolarization-activated cyclic nucleotide-gated cation channel [172] [173]
*Pueraria lobata (Willd) Ohwi*, *Pueraria thunbergiana Benth*.	Puerarin	Antiarrhythmia (Wei et al., 2015 [175]), anti-ischemic [176], antihypertensive [177], lowering blood lipid [178], lowering blood sugar [179], expanding coronary arteries [228], and anti-inflammatory [181]	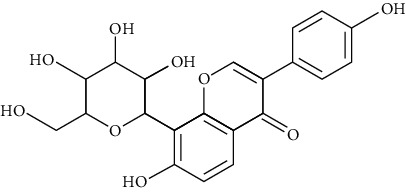	Delayed rectifier potassium channel, inward rectifier potassium channel [185] [186], calcium-activated potassium channel [187]	L-type calcium [188]	Voltage-gated cardiac sodium channel [191]		
*Saussurea involucrata* (*Kar. et Kir*.) *Sch*.-*Bip*.	Acacetin	Antiarrhythmic [193], antitumor, anti-inflammatory, antifibrosis, antioxidation [198]	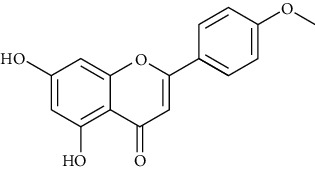	Ultrarapid delayed rectifier potassium channel and the transient outward potassium channel [193], hERG, small conductance Ca2+-activated K+ channels [204], transient outward potassium channel [200]				
*Glycyrrhiza uralensis Fisch.*, *Glycyrrhiza inflata Bat*., or *Glycyrrhiza glabra L*.	Glycyrrhizic acid	Antiarrhythmia [205], antioxidant, anti-inflammatory [206], antiviral [229], antitumor (Zuo et al., 2021)	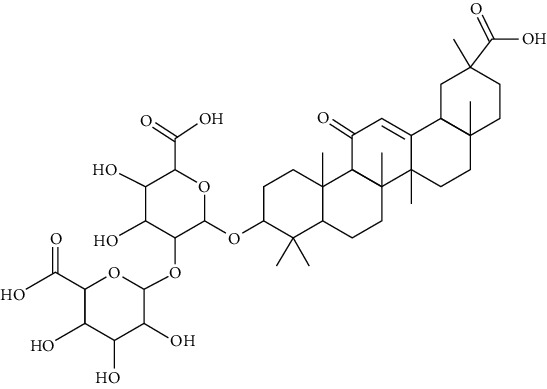		L-type calcium channel [205]	Voltage-gated cardiac sodium channel [210] [211]		
*Artemisia carvifolia Buch.-Ham. ex Roxb. Hort. Beng.*	Artemisinin	Antimalarial [212], antitumor [213], anti-inflammatory [214], antiviral [215], antibacterial [216], and antifibrotic [217]	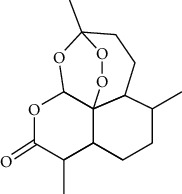	Delayed rectifier potassium channel, inward rectifier potassium channel, transient outward channel [218] [219]	L-type calcium channel [220]		Cx43[221] [222]	Hyperpolarization-activated cyclic nucleotide-gated cation channel [223]

**Table 2:** Natural product active components' regulatory effects on the genes responsible for ion channel proteins.

Gene	Encoding protein	Upregulate	Downregulate
SCN5A	Nav1.5		GFA
KCNB1	Kv2.1	Matrine	
KCND3	Kv4.3	PNS	
KCNE1	Mink		Tanshinone IIA
KCNH2	Kv11.1	Matrine	Berberine, matrine, resveratrol
KCNJ2	Kir2.1	Matrine, tanshinone IIA	
KCNQ1	Kv7.1		Tanshinone IIA
KCNN3	KCa2.3	PNS	
hHCN4	Hyperpolarization-activated cyclic nucleotide gated potassium channel		Berberine
SK3	Small conductance calcium activates potassium channel 3	PNS	
CACNA1C	Cav1. 2	Matrine, Tanshinone IIA	PNS
CACNB1	Calcium voltage-gated channel auxiliary subunit beta		PNS
RyR2	Ryanodine receptor		Berberine
GJA1	Gap junction protein 43 (Cx43)	Resveratrol	
GJA5	Gap junction protein 40 (Cx40)	GFA, puerarin	
ATP2A2	ATPase sarcoplasmic/endoplasmic reticulum Ca2+ transporter		PNS, berberine

## Data Availability

The data used to support the findings in this study are included within the article.

## Abbreviations

AAD: Antiarrhythmic drugs
CNKI: Chinese National Knowledge Infrastructure
AF: Atrial fibrillation
NICE: National Institute for Health and Care Excellence
AHA: American Heart Association
ACC: American College of Cardiology
HRS: Heart Rhythm Society
EACTS: European Association of Cardio-Thoracic Surgery
KCNA5: Potassium voltage-gated channel subfamily A member 5
KCNN3: Potassium calcium-activated channel subfamily N member 3
miR-499: MicroRNA-499
miR-29: MicroRNA-29
IKr: Delayed rapid-rectifying potassium current
IKM_3_: M3 receptor-mediated K^+^ current
CICR: Calcium-induced calcium release
SCN5A: Sodium voltage-gated channel alpha subunit 5
KCNB1: Potassium voltage-gated channel subfamily B member 1
PI3K: Phosphatidylinositol 3 kinase
KCNE1: Potassium voltage-gated channel subfamily E member 1
CACNB1: Calcium voltage-gated channel auxiliary subunit beta 1
GJA1: Gap junction protein alpha 1
GFA: Guanfu base A
Iks: Slow activated delayed rectified potassium current
KCNQ1: Potassium voltage-gated channel subfamily Q member 1
TGF-*β*1: Transforming growth factor beta 1
INCX: Reverse sodium-calcium exchange current
CaM: Calmodulin
IK1: Inward rectifier potassium channel
RyR2: Ryanodine receptor2
SERCA2: Sarcoplasmic reticular Ca^2+^-ATPase
KCNE1: Potassium calcium-activated channel subfamily E member 1
KCNE3: Potassium calcium-activated channel subfamily E member 3
HCN2: Hyperpolarization-activated cation channel 2
KChIP2: Potassium channel interacting protein 2
PNS: Panax notoginseng saponins
APD: Action potential duration
ERP: Effective refractory period
SK3: Small conductance calcium activates potassium channel 3
ATP2A2: ATPase sarcoplasmic/endoplasmic reticulum Ca^2+^ receptor 2
HERG: Human ether-a-go-go-related gene
hHCN4: Hyperpolarization-activated cyclic nucleotide-gated 4
APA: Action potential amplitude
KCNH2: Potassium voltage-gated channel subfamily H member 2
KCND3: Potassium voltage-gated channel subfamily D member 3
KCNJ2: Potassium voltage-gated channel subfamily J member 2
KCNQ1: Potassium voltage-gated channel subfamily Q member 1
CACNA1C: Calcium voltage-gated channel subunit alpha1 C
RyR2: Ryanodine receptor 2
GJA5: Gap junction protein alpha 5
Dau: Dauricine
Ikr: Rapid activated delayed rectified potassium current
TSP-1: Thrombospondin-1
Ang II: Angiotensin II
CaMK II: Calmodulin-activated protein kinase II
Cx43: Connexin 43
SIRT1: Sirtuin 1.
